# Community surveillance and response to maternal and child deaths in low- and middle-income countries: A scoping review

**DOI:** 10.1371/journal.pone.0248143

**Published:** 2021-03-16

**Authors:** Tariro J. Basera, Kathrin Schmitz, Jessica Price, Merlin Willcox, Edna N. Bosire, Ademola Ajuwon, Marjorie Mbule, Agnes Ronan, Fiona Burtt, Esca Scheepers, Jude Igumbor

**Affiliations:** 1 School of Public Health, Faculty of Health Sciences, University of the Witwatersrand, Johannesburg, South Africa; 2 mothers2mothers, Cape Town, South Africa; 3 MRC-Wits Rural Public Health and Health Transitions Research Unit (Agincourt), School of Public Health, Faculty of Health Sciences, University of the Witwatersrand, Johannesburg, South Africa; 4 School of Primary Care, Population Sciences and Medical Education, University of Southampton, Southampton, United Kingdom; 5 South African Medical Research Council Developmental Pathways for Health Research Unit (DPHRU), School of Clinical Medicine, Faculty of Health Sciences, University of the Witwatersrand, Johannesburg, South Africa; 6 Department of Health Promotion and Education, Faculty of Public Health, University of Ibadan, Ibadan, Nigeria; Medical Research Council, SOUTH AFRICA

## Abstract

**Background:**

Civil registration and vital statistics (CRVS) systems do not produce comprehensive data on maternal and child deaths in most low- and middle-income countries (LMICs), with most births and deaths which occur outside the formal health system going unreported. Community-based death reporting, investigation and review processes are being used in these settings to augment official registration of maternal and child deaths and to identify death-specific factors and associated barriers to maternal and childcare. This study aims to review how community-based maternal and child death reporting, investigation and review processes are carried out in LMICs.

**Methods:**

We conducted a scoping review of the literature published in English from January 2013 to November 2020, searching PubMed, EMBASE, PsycINFO, Joanna Briggs, The Cochrane Library, EBM reviews, Scopus, and Web of Science databases. We used descriptive analysis to outline the scope, design, and distribution of literature included in the study and to present the content extracted from each article. The scoping review is reported following the PRISMA reporting guideline for systematic reviews.

**Results:**

Of 3162 screened articles, 43 articles that described community-based maternal and child death review processes across ten countries in Africa and Asia were included. A variety of approaches were used to report and investigate deaths in the community, including identification of deaths by community health workers (CHWs) and other community informants, reproductive age mortality surveys, verbal autopsy, and social autopsy. Community notification of deaths by CHWs complements registration of maternal and child deaths missed by routinely collected sources of information, including the CRVS systems which mostly capture deaths occurring in health facilities. However, the accuracy and completeness of data reported by CHWs are sub-optimal.

**Conclusions:**

Community-based death reporting complements formal registration of maternal and child deaths in LMICs. While research shows that community-based maternal and child death reporting was feasible, the accuracy and completeness of data reported by CHWs are sub-optimal but amenable to targeted support and supervision. Studies to further improve the process of engaging communities in the review, as well as collection and investigation of deaths in LMICs, could empower communities to respond more effectively and have a greater impact on reducing maternal and child mortality.

## Introduction

Despite efforts to improve healthcare delivery, maternal and child mortality remain high in many low- and middle-income countries (LMICs). Approximately 830 women die each day globally during or following pregnancy and childbirth, with over 90% of these deaths occurring in LMICs [1]. Sub-Saharan Africa and the South Asian regions account for 66% and 33% of total global maternal deaths, respectively [2]. Similarly, under-5 mortality remains highest in sub-Saharan Africa, which accounts for half of all child deaths globally, and central and southern Asia, where 30.4% of all under-5 deaths occur [3]. In 2017, an estimated 5.5 million children under five years old died, of whom 2.5 million died in the first month of life [4].

High maternal and child mortality are attributed partly to poor quality of maternal and newborn care resulting from many factors, including delays in seeking and receiving care, delays in referrals for treatment, stock-outs of essential commodities, poor monitoring during labour, inadequate skills for providing emergency obstetric and neonatal care and managing other co-infections, lack of medical supplies including blood, widespread understaffing, and home birth [5, 6].

The implementation of interventions to reduce maternal and child mortality requires an adequate understanding of the causes of deaths and barriers to healthcare for mothers and children [7]. Creation of functional CRVS systems and quality data collected at the community level are critical to achieving this goal [8]. In LMICs, the ability to identify barriers and develop strategies to overcome them is often constrained by the lack of good quality data on maternal and child deaths from routinely collected sources of information [9]. For example, much of the available data for tracking births and deaths in LMICs is mainly from health facilities (hospitals) [10, 11]. All births and deaths should be registered through national CRVS systems in LMICs; however, this is difficult to achieve in LMIC settings because a significant number of births and deaths occur outside formal health systems [12, 13]. In 29 sub-Saharan African countries, for example, 77.7% of births occurred outside health facilities [14], while approximately 50% of under-five deaths occurred at home [15]. A comprehensive tracking system for maternal and child healthcare would integrate both facility- and community-based sources of data to inform interventions aimed at reducing maternal and child mortality in LMICs.

Community-based data sources include household surveys, verbal autopsies, social autopsies and demographic surveillance systems aimed at identifying maternal and child deaths [10, 16–18]. A verbal autopsy (VA) involves a structured interview with the caregiver of the deceased, which aims to establish the probable biological cause of death [8]. The WHO has produced standardised VA questionnaires which elicit information on symptoms experienced during the final illness, the deceased’s medical history and, where relevant, details related to any pregnancies, antenatal care, labour and delivery [19]. According to Kalter and colleagues [20], a social autopsy (SA) refers to “an interview aimed at identifying social, behavioural, and health system contributors to death both within health facilities, and at different stages of the patient pathway.” Community-based death surveillance and response (CDSR) is a process of active case-finding that starts with notification of a maternal or child death by field-level health workers or community informants, investigation of the causes of deaths, relaying of this information to a focal person at the district-level health facility, and involving community members in review and response to prevent similar deaths in the future [7]. Community-based data collection methods have the potential to augment and improve overall data on maternal and child deaths in LMICs.

VAs and SAs have been used widely across LMICs [21]. However, less is known about how these data collection tools feed into implementation of responses to maternal and child deaths in LMICs and how they can be used to help decision-makers identify modifiable factors that contribute to deaths and the development of local interventions to reduce mortality. To inform the improvement of maternal and child death surveillance and response, therefore, this paper provides findings of a scoping review on community-based surveillance and response to maternal and child deaths in LMICs.

### Study purpose

The scoping review aimed to explore the concept and current practice of community-based maternal and child death surveillance and response in LMICs. The key objectives were:

- To describe the methodology for CDSR of maternal and child deaths and investigation of causes of death by community health workers in LMICs.
- To determine the outcomes of CDSR processes for maternal and child deaths in LMICs.

## Methods

A scoping review was conducted using Arksey and O’Malley’s framework [22], and the findings are presented using a narrative synthesis.

### Criteria for inclusion

Studies were included if they reported on the investigation of maternal deaths and/or deaths of children under five years (including neonatal deaths) in LMICs. Studies had to include maternal and/or child deaths in the community alone or together with deaths in health facilities. Studies on facility-based deaths alone were excluded as they did not capture deaths in the community. Studies did not have to include a component of community-based review or response to the deaths. We included both qualitative and quantitative studies published in the English language from January 2013 to November 2020.

### Search strategy

Our search strategy was designed to concentrate on deaths identified through CHWs or similar lay health worker cadres, following which verbal autopsy and social autopsy (VASA) were conducted to investigate the biological causes of, and avoidable factors contributing to, maternal and child deaths. We searched eight databases, including PubMed, EMBASE, PsycINFO, Joanna Briggs, The Cochrane Library, EBM reviews, Scopus, and Web of Science for articles published between January 2013 and November 2020, using Mesh Headings and free-text keywords that applied to community-based maternal and child death reviews. The timeframe was limited to literature published between January 2013 and November 2020 to gain information on current practice following the 2011 comprehensive review on social autopsy for maternal and child deaths by Kalter and colleagues [20]. We used the following key search terms combined using the Boolean operators ‘AND” and “OR”: “verbal autopsy”, “social autopsy”, “death review”, “post mortem interview”, “death surveillance and response”, “MPDSR”, “MDSR”, “confidential enquiry”, “death reporting”, “death registration”, “death investigation”, “death ascertainment”, “maternal deaths”, “under-five mortality”, “infant deaths”, “neonatal deaths”, “perinatal deaths”, “lay health worker”, “community health worker”, “community informant”, “community-based” (S1 Table). References from included articles were hand-searched for additional studies.

### Data extraction and analysis

Following our search, all identified articles were collated and uploaded into EndNote and exported to Covidence software for screening, where duplicates were removed. Two reviewers independently evaluated the title and abstract of each article for assessment against the inclusion criteria. To determine texts included in the review, we conducted duplicate full-text screening. Any disagreements between the reviewers were resolved through discussions among the review team.

A standardised data evaluation tool from the Joanna Briggs Institute (JBI) Reviewers’ Manual was used to extract and synthesise data [23]. The data obtained included the following: Details of the processes and procedures of reporting, investigation and review of maternal and child deaths; measures to improve validity and data quality; the role of community health worker cadres/community informants in the death surveillance and response process; profile of respondents/participants; results or outcomes of the CDSR, including the number of maternal and child deaths notified and causes of death identified; barriers and facilitators to a successful CDSR; and perceptions and experiences of people involved in the process (S2 Table).

We used narrative synthesis to describe the scope, design, and distribution of literature included in the review and to present the contents extracted from each article [22]. The scoping review is reported following the PRISMA reporting guideline for systematic reviews [24].

## Results

**Fig 1:**
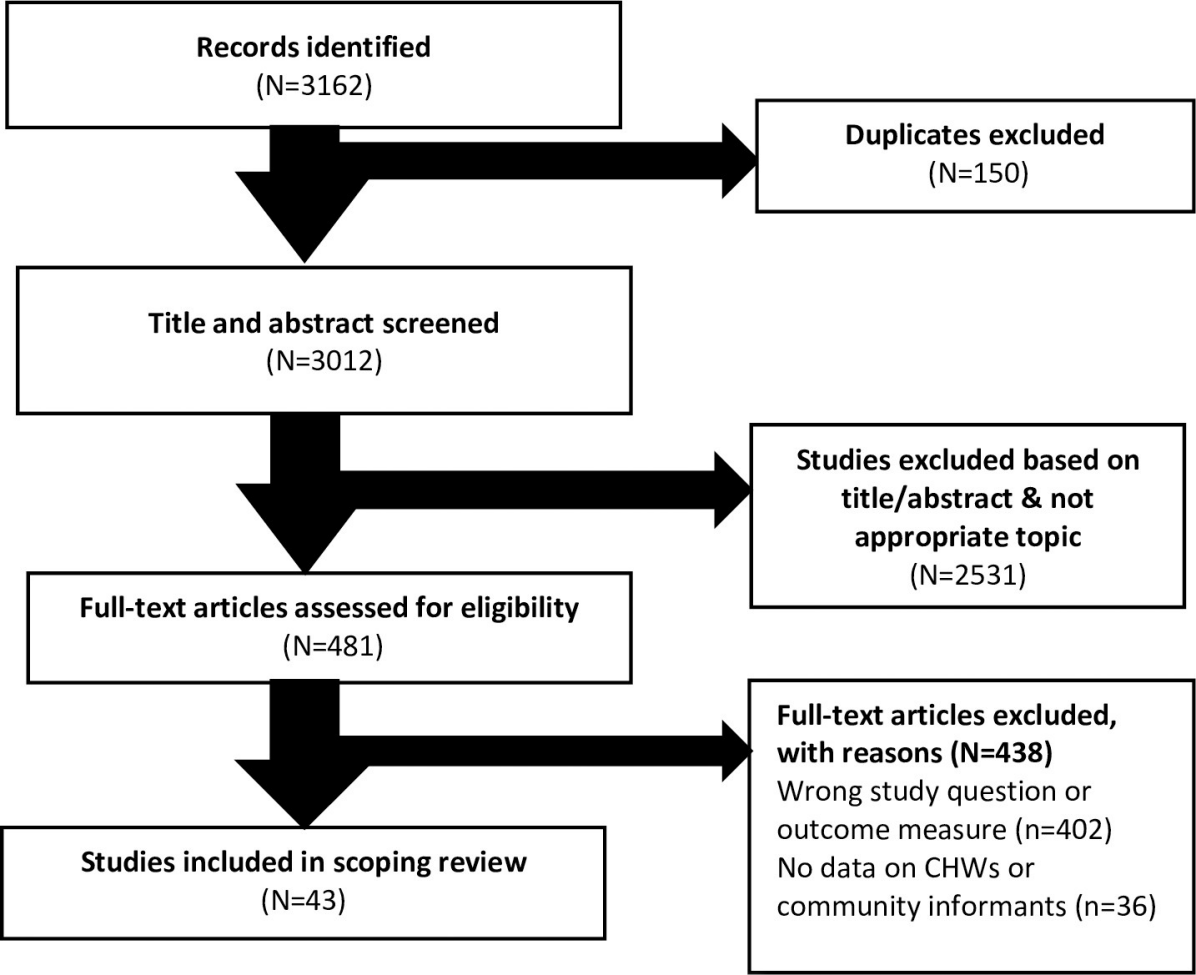
PRISMA flow diagram of literature search strategy.

The initial search strategy identified 3162 articles. Following exclusion of all duplicates, we screened 3012 titles and abstracts and eliminated 2531 because they fell outside the scope of interest of the study. We reviewed the full text of the remaining 481 articles, of which 43 were included in this review. The Prisma flow chart in Fig 1 illustrates the selection process and number of articles included or excluded at each stage of the selection process.

### Descriptive information for included studies

Of the 43 selected articles, 23 reported on the deaths of children under five years, 13 reported on maternal deaths, and seven covered both maternal and child deaths. Most studies (n = 28, 65.1%) were from sub-Saharan Africa, with Malawi having the highest number (Malawi n = 5). Fifteen articles (34.9%) were from Asian countries; the majority from India (n = 7) (Table 1).

**Table 1:** Summary of characteristics and methods used to identify deaths, and the number of deaths identified.

Source	Persons responsible for reporting deaths	Method for death reporting (paper, phone, etc.)	Persons responsible for investigating	Method for investigating & tools	Time frame for investigation	No. of deaths reported	No. of deaths investigated
**Maternal deaths**							
Dikid et al. [25] India	Accredited social health activists (ASHAs) & Anganwadi workers investigate community reports of suspected maternal deaths	Not specified	Government staff investigated deaths using verbal autopsy (VA)	VA Maternal death enquiry questionnaire	March to December 2009	1120 maternal deaths were identified in four states	1103 (98.5%) of the identified maternal deaths were investigated
Singh et al. [44] India	Frontline health workers routinely collect and notify maternal deaths to the primary healthcare medical officer within 24 hours	Line list of maternal deaths	Senior Public Health Officer conducted the VA	VA Government of India standardised VA forms	April to September 2012	284 maternal deaths were recorded	193 (68%) maternal deaths were reviewed
Halim et al. [37] Bangladesh	Family welfare assistants and health assistants visit households to confirm the death of any woman known to be pregnant who died	Informants completed a notification slip	Health inspectors and family inspectors conducted VA	VA Modified WHO VA tool	January 2011 to January 2012	571 maternal deaths identified in four districts	VA performed for all 571 deaths (100%)
Biswas et al. [38] Bangladesh	Family welfare assistants and health assistants visit households to confirm the death of any woman known to be pregnant who died	Health Assistants and Family Welfare Assistants completed a community death notification slip	Health Inspector, Assistant health inspector and family planning inspector conducted VA interviews	VA Modified WHO VA tool	January to March 2018	34 maternal deaths were reported	VA performed for all 34 maternal deaths
Mir et al. [26] Pakistan	Lady health workers and religious leaders collected information related to deaths in women	Women of reproductive age death listing form	MADE-FOR study team	VA Revised WHO VA questionnaire	January 2012 to December 2013	In Chikwal, 62 pregnancy-related deaths identified by lady health workers, 38 by religious leaders & 28 by both. In Talagang, 37 deaths were identified by lady health workers, 26 religious leaders & 18 by both	VA was performed for 1808 (90.3%) deaths out of the 2001 deaths identified
Naik et al. [78] India	Health workers record and notify maternal deaths routinely to the Medical Officer	Line list of maternal deaths	Senior Public Health Officer conducted the VA	VA Government of India standardised VA forms	August-November 2014	67% of deaths investigated through district meetings	VA performed for 22 maternal deaths
Moshabela et al. [40] Senegal	CHWs identified maternal deaths through active household-level surveillance of pregnancies, births & deaths	mHealth platform Childcare+	Trained fieldworker conducted the verbal & social autopsy (SA) interview	VASA MVP VASA 00tool	January 2007 to December 2012	Two maternal deaths were identified in 2007, 2 in 2008, 1 in 2009, 3 in 2010, 5 deaths in 2011 and 2 in 2012	Five maternal deaths were investigated
Bayley et al. [31] Malawi	Community team members (Health Surveillance Assistants (HSAs), group village headman & volunteers) identify & notify a maternal death in the community	Death review form	Community team	VA Community VA form	July 2011 to June 2012	52 maternal deaths were identified–of these 25 (48%) were detected by the existing notification system at the district hospital, the community team identified 43 (83%) including 27 more (52%) which were unreported at the hospital	45 maternal deaths (86.5%) were investigated
Adomako et al. [32] Ghana	Community-based surveillance volunteers identified deaths of women of reproductive age and administered RAMOS 4+2 interviews	RAMOS interview record sheets	A community health nurse performed the VA	VA Modified WHO VA form	May to August 2013	132 deaths of women of reproductive age identified through RAMOS. VA found 64 maternal deaths. Identified 13 more deaths that occurred at home which were not included in the facility records	118 deaths (89.4%) investigated using VA
Mgawadere et al [27] Malawi Mgawadere et al. [28] Malawi	Death of women aged 15–49 years were identified by: Health providers; Heads of households; Village leaders; Traditional healers; HSAs; Burial sites; Village registers; Traditional birth attendants and Police	Deaths of women of reproductive age (WRA) were reported in writing or by telephone to the research staff based at the district hospital	Trained research staff performed VA	VA WHO VA tool	1 December 2011 to 30 November 2012	424 deaths of women of reproductive age were identified, 151 were maternal deaths. Of these, only 86 had been recorded via the health management information system–all of which were facility-based deaths	All 151 deaths investigated using VA
Zaba et al. [52] Kenya, Tanzania, Uganda, Malawi, Zimbabwe, South Africa	Community-based informants notified pregnancy-related deaths at Masaka (Uganda) & Manicaland (Zimbabwe) while at all sites in Kenya, Tanzania, Malawi & South Africa, VAs were triggered by reports of deaths collected during demographic surveillance	Paper-based reporting	Not specified	VA	Between June 1989 and April 2012	235 pregnancy related deaths identified. 40 (17%) were identified as pregnancy-related by both VA and demographic surveillance data. 144 were identified as pregnancy-related based on VA reports alone (61.3%). The remaining 51 (21.7%) were identified through demographic surveillance alone	VA was performed for 184 deaths (78.3%)
Akosah & Dapaah [80] Ghana	CHWs registered household members within their community including pregnant women & children under -five years	Data was sent via text messages to a central server	VA specialists conducted in-depth VA investigations	Not specified	2010 to 2014	160 deaths of children under five reported and 1 maternal death	All deaths were investigated
Gilmartin & Levin [59] Burkina Faso	Health agents identified & notified maternal deaths	Reported via mobile phone	Health agents investigated causes of maternal deaths	Not specified	2012 to 2014	1746 maternal deaths have been reported to the national level	63.2% of maternal deaths were audited in 2014
**Child deaths**							
Rai et al. [42] India	ASHAs identify infant deaths during routine home visits	Not specified	A health worker performed the VA, and a medical doctor administered a SA	VASA Ballabgarh VA tool INDEPTH-WHO SA tool	From 2008 to 2012 SA conducted between 1 January 2012 & 31 June 2012	514 infant deaths reported	All 514 infant deaths (100%) were investigated using VA. 91 deaths (17.7%) were investigated using SA
Shikha et al. [77] India	ASHAs, Family health workers & Anganwadi workers notified the concerned primary healthcare Medical Officer of an infant’s death	Telephonically and sends a primary informant form filled within 24 hours to the relevant Medical Officer	Medical officers of each primary health centre conducted VA	VA VA form	2012–2013	345 infant deaths	302 VA forms were available (87.5%)
Kakoty et al. [46] India	Anganwadi workers reported deaths	Meeting with Anganwadi workers	Not specified	VA VA questionnaire	1 January to 31 March 2016	Not specified	90 neonatal deaths (11.5%) were purposively selected for VA
Soofi et al. [9] Pakistan	CHW identified deaths	CHWs recorded information on the VA questionnaire	CHWs conducted VA	VA WHO/LSTMH/John Hopkins University modified VA tool 2000	August 2006 –February 2008	784 neonatal deaths identified	VA conducted for 626 neonatal deaths (79.8%)
Willcox et al. [39] Mali & Uganda	Village Health Teams reported incidents of child deaths to the study team	Deaths reported by mobile phone to a fieldworker at the sub-district level	A fieldworker performed the VASA	VASA Modified QUARITE questionnaire	August-October 2011 & 2012–2014	762 deaths of children under five years were identified in Mali and 442 in Uganda	VA done for all deaths (100%)
Nabukalu et al. [50] Uganda	Village Health Teams (CHWs) & village council chairman identified deaths in households	VA questionnaire	Trained VHT of that respective village conducted VA interview	VA WHO 2014 VA questionnaire	1^st^ of January 2016 to 31^st^ December 2016	230 deaths identified (53.5% were not recorded in the facility-based surveillance system), 77 among children under five years	VA conducted for all identified deaths (100%)
Hutain et al. [35] Sierra Leone	CHWs registered under-five child deaths during routine monthly household visits	Paper forms issued by the Ministry of Health	The VA Officer (clinician) located families of the deceased with the help of the reporting CHW and Community Development Officer and conducted VAs	VA WHO 2007 VA tool; WHO 2014 VA tool; Population Health Metrics Research Consortium Shortened Questionnaire	October 2015 to May 2017	CHWs reported 582 deaths of children under five, 243 of these were recorded in the vital events database	VA was conducted for 222 deaths (38.1%)
Gupta et al. [47] Rwanda	Deaths identified through facility registers, CHW reports, CHW-held community death records, & phone-based reporting system	CHW report & death records	Trained data collectors performed VA	VA 2012 WHO VA tool	1 March 2013 to 28 February 2014	618 deaths of children under five years, of which 174 were neonatal deaths	All deaths were investigated (100%)
Bogale et al. [6] Ethiopia	Community data collectors record death in the 28 days of life as part of the ongoing Health & Demographic Surveillance System	VA forms	HDSS supervisors & trained data collectors conducted SA for deaths identified through VA	SA INDEPTH Network SA tool	October 2013 to September 2017	VA identified 39 neonatal deaths	SA was conducted for 37 neonatal deaths (94.9%)
Kallander et al. [51] Mozambique	CHWs visit & record all vital events including child deaths & report in their monthly report	Monthly reports	Research assistant investigated the circumstances of the death	VASA WHO VA tool; INDEPTH network SA tool	1 January to 31 December 2015	117 deaths of children under five years	VA was conducted for 115 deaths (98.3%)
Roder-Dewan et al. [45] Rwanda	Deaths were identified through health records, Ministry of Health reporting systems & Monitoring of Vital Events using Information Technology by CHWs	CHWs reported vital events telephonically	Trained interviewers conducted interviews	VASA 2012 WHO VASA tool; Rwanda Ministry of Health Death Audit tool	March 2013 to February 2014	259 deaths of children under five	77 VAs were analysed (29.7%)
Igumbor et al. [41] South Africa	Deaths were identified by community health workers (Mentor Mothers) based on information obtained from street committee members and facilities	Community health workers captured information on smartphones (REDCap software)	Trained CHWs conducted VASA interviews	VASA VASA questionnaire	January 2017 to July 2019	19 neonates and infant deaths and 3 maternal deaths	19 deaths were investigated using VASA (86.4%)
Keenan et al. [79] Niger	Deaths were identified through house-to-house censuses	Child deaths notified through household censuses done every 6 months	VA interviews were done by Medical Officers and Field Personnel	VA 2007 WHO VA questionnaire	26 May to 17 May 2018	3615 deaths of children aged 1–59 months	3301 VAs performed (91.3%)

Thirty-five studies were prospective enquiries while four were retrospective in design and the other four were cross-sectional studies. The recall period was up to eight weeks in seven studies, two years in four studies, while one study retrospectively investigated deaths in five years. The recall period was not reported in eleven studies (S2 Table).

### Mechanisms for identifying and reporting maternal and child deaths in the community

#### Identifying maternal and child deaths

*Community-based informants*. Although different terminologies were used to describe community health workers, they performed similar roles in tracking and collecting information on vital events, including maternal and child deaths, by visiting homes or other community settings. Volunteers also identified maternal and child deaths from civil society organisations and non-governmental organisations (e.g. White Ribbon Alliance working in Orissa State, India [25], religious leaders [26], traditional healers, burial records, village registers and police stations [27, 28], and village headmen [29–31].

*Reproductive Age Mortality Study (RAMOS)*. In three studies, maternal deaths were identified using RAMOS, which triangulates data from multiple records of maternal deaths. Sources include vital registration, patient records, interviews with family members, coroner’s office, the police, and traditional birth attendants [27, 28]. Once all maternal deaths are identified, a VA was undertaken for all deaths to ascertain the cause(s) of each death. One study in Ghana showed that community informants administered the shorter version of the RAMOS questionnaire, with four ‘yes’ or ‘no’ questions to identify a maternal death among women of reproductive age. Community health nurses and midwives then conducted VAs at households in which an affirmative answer had been given to at least one of the four questions [32].

As illustrated in Table 1, CHWs demonstrated capacity to identify maternal and child deaths, particularly those missed by health facility-based surveillance systems because they did not occur at facilities.

#### Accuracy and completeness of CHW reporting of maternal and child deaths

Accuracy and completeness of CHW reporting systems are a crucial component of CDSR systems. Only four studies reported on this issue, from Malawi [33], Ethiopia [34], Sierra Leone [35], and Mali [36]. In Mali, for example, the under-five mortality rate calculated from CHW data was not different from the rate estimated using household surveys, with a difference of one death per 1000 live births [36]. However, CHW still missed some vital events. For example, in Malawi, compared to data obtained from a retrospective pregnancy history, Health Surveillance Agents (government employed CHWs) missed 33% of births and deaths [33]. Also, 90.5% of under-five deaths were reported by the Health Surveillance Agents in phase one of the Real-Time Monitoring of Under-Five Mortality project (January 2010 to August 2012), but this dropped to 87% in the second phase (October 2011 to February 2012) [33]. Reasons for decline in death reporting included turnover of CHWs, a decrease in the level of supervision at the district level, and delays in the transfer of data forms between CHWs, their supervisors and the district manager [34]. In Sierra Leone, the register for child deaths was incomplete because only about 40% of CHWs submitted a monthly report of under-five deaths between 2014 and 2017 [35].

#### Quality assurance methods

Different quality assurance methods to enhance the validity of reporting of community-based maternal and child deaths were applied. Families were revisited in case of any inconsistencies to clarify inaccuracy or incompleteness of data in Bangladesh [37] and the MPDSR Quality Improvement committee checked 10% of the case studies to ensure quality and consistency of the VA data [38]. In Mali and Uganda, a random sample of VAs was re-investigated to cross-check with other sources of information to ensure the accuracy of the information supplied by community informants and fieldworkers [39]. Childcount+ platform, a mHealth application with built-in reminders, was used to collect data and monitor the performance and amount of work done by CHWs in Senegal [40]. In Pakistan and Senegal refresher training was provided for community informants [9, 40]. In Malawi, each CHW was assigned a supervisor who monitored the accuracy and completeness of the village registers completed by CHWs. The supervisor made a correction where necessary, provided immediate feedback to the CHWs, and conducted on-the-spot retraining for CHW [34]. In South Africa, completion of post-interview reflections by CHWs and conducting debriefing sessions were integrated into the CLMDR as quality assurance tools for monitoring, evaluating and responding to field experiences [41].

#### Methods for investigating causes and modifiable factors that contributed to maternal and child deaths

The primary method of investigating biological causes of death is VA, while SA is used to investigate circumstances of death including care-seeking pathways. Table 1 illustrates the instruments used to conduct a verbal autopsy and social autopsy (VASA). Six of the studies did not specify the source of tools used for conducting VASA. Of the remaining studies, the majority (eleven) of the investigators adopted the WHO tools for VA and SA. Other less frequently used tools are INDEPTH Network’s VASA tool [6, 42], the QUARITE Trial tool [39], the Millennium Village Project (MVP) standardised tool [40], the Ballabgarb tool [42] and the Population Health Metrics Research Consortium shortened questionnaire [35]. There is generally a lack of coherence in the domains, length and type of questions in the different VASA instruments used in the reviewed studies.

*Verbal autopsy*. VA was used to obtain information about the cause of death by interviewing the deceased’s family, including the husband, mother, sister, mother-in-law, sister-in-law, or a traditional birth attendant who had provided care to the deceased [21, 43]. One study did not mention the respondents in VA interviews [44].

VA interviews were conducted within 14 to 30 days of the death in prospective enquiries, giving the family adequate time to grieve [42, 45, 46]. The recall period was longer in retrospective studies–ranging from three weeks in Rwanda [47] and two years in Pakistan [26] to five years in Ghana [32]. A recall of up to one year does not seem to affect the validity of the findings. A study conducted in India and Philippines reported that the probability of a correct diagnosis in VAs collected 3–11 months after death would, on average, be 95.9% of that in VAs collected within three months of death [48]. In another study, the probability of VAs to assign a cause of death decreased by 0.55% per month in the period after death [49].

There was variation in the type of personnel who conducted VAs in the studies where this information was provided. CHWs administered VAs in three studies, in Pakistan, Malawi and Uganda [9, 31, 50], with varying levels of education. In Pakistan, the CHWs who conducted the VAs were educated to at least college level [9], while the CHWs who administered VAs in Uganda had at least one year of secondary education [50]. The level of education of the Health Surveillance Assistants in Malawi was not specified [31].

The methods for determining the cause of death varied among the studies. Some used “physician review”, whereby physicians, obstetrician-gynaecologists, paediatricians and midwives independently assessed data from the interviews to assign a single cause of death based on the International Classification of Diseases [6, 9, 27, 28, 37]. Other studies used a computerised algorithm (InterVA-M software) to determine the most likely medical cause of death [25, 26, 35, 40, 45, 47, 50, 51], or analysis of data by researchers [42, 52].

*Social autopsy*. SA was conducted in ten studies. In India [40], Senegal [39], Uganda and Mali [39], Mozambique [51] Rwanda [45] and South Africa [41], the SA was administered together with a VA. When frontline government health workers in Bangladesh receive notification about the death of a mother or newborn, they conduct a SA and a community death review [7, 16, 53].

#### Methods for review and response to deaths

Community death review meetings and confidential enquiries which include the participation of lay health workers, some community members and community leaders, are used to discuss remedial actions or strategies to prevent future deaths in the community and facilities [7, 31].

*Community death review meeting*. Community death review meetings were reported in five studies (Table 2). The community meeting involved asking for the potential cause(s) of death and avoidable factors, as well as to propose actions to prevent future deaths in Malawi [31], Sierra Leone [54], Mali and Uganda [39], Senegal [40] and Bangladesh [7]. In Sierra Leone, local community leaders, CHW supervisors and health centre staff were actors in community health data reviews [35, 54]. In Bangladesh, community death review meetings have on average 40–50 members, including health workers, community informants, and community members [18]. In Malawi, the public meeting was attended by representatives from the district hospital and health centre [31]. The CHWs presented a summary of the case of maternal death with the family’s consent. The community made recommendations and agreed on community factors that contributed to maternal mortality. In turn, the health workers reported proposed strategies to prevent future deaths, and community members questioned them about whether their action points from previous meetings had been completed [31]. In general, the review process was highly acceptable to the community, with communities taking ownership of the capturing of deaths and the response to address avoidable factors of maternal and child deaths. Feedback was reported to the community to enhance their involvement and increase accountability of healthcare workers to the community they serve [16, 31].

**Table 2:** Summary of studies where a community death review was conducted.

Source	Person(s) responsible for review or response	Method(s) for review & response	Number of deaths reviewed	Response/ recommendations implemented
Biswas et al. [7] Bangladesh	• Health inspectors, Assistant Health Inspector & Family Planning Inspector were responsible for facilitating reviews • A community representative chaired each meeting	• Community discussion attended by 20–50 community members • Field notes taken during the discussions following a guideline	SA was performed for 28 out of 59 maternal deaths notified between January & December 2010	• Regular antenatal care visits • Mothers to prepare birth planning & ensure they deliver their baby at the health facility by trained provider • Community awareness of maternal complications
Bayley et al [31] Malawi	• HSAs discussed factors contributing to maternal deaths in a meeting held in the community • HSAs reported the information from the VA and the community team discussions • HSAs summarised the case & facilitated an open discussion	• Community team meeting • Meeting at the local health facility/district hospital • Public meeting in the local community	• 37 deaths were discussed at a community death review meeting • 44 deaths were discussed at a health facility death review meeting • 32 deaths were discussed at the community feedback meeting	• Improving drug supplies–adequate stock of antihypertensive medication • Training sessions for clinicians • Health education events for communities on maternal health topics • Improved provision of emergency transport • Changing protocols to improve access to rural hospitals
Willcox et al. [39] Mali & Uganda	Online and face-to-face meetings chaired by local paediatrician with 8–15 attendees (local doctors, nurses, health-care assistants & one community representative [VHT members in Uganda & local traditional health practitioners in Mali])	• Confidential enquiry (District panel meeting) • Community meeting (Whole-village meetings in Mali & meetings of VHTs in Uganda)	762 deaths in Mali and 442 in Uganda were reviewed	• Community education & improving treatment seekingEstablishment of loan fund in Mali to facilitate emergency access to healthcare • Transfer of staff to understaffed health centres • Mobile phone calling circle to solve a staff communication problem • Refresher training for local staff • Introduction of clinical guidelines
Moshabela et al. [40] Senegal	Regional Millennium Village Project (MVP) staff	Monthly community meetings with regional MVP staff & local community members to discuss deidentified VASA findings alongside other routine health indicators	5 maternal deaths	• Provision of surgical packs, equipment & drugs by MVP • Reshuffling, training & supervision of the surgical team by the hospital • Combined effort to track, avail and prepare blood donors for obstetric emergency care • Quarterly mortality reviews • Overhaul of the hospital infrastructure
O’Conner et al. [54] Sierra Leone	• The Health Management Committee Chair for the community chaired the meeting • Sessions were facilitated by operations research study staff. Over time, participants took the lead in reviewing the data and reporting the findings	Community health data review meetings attended by 30 to 50 people	CHWs submitted 2409 reports over 34 months which were reviewed in 29 meetings from July 2015 to April 2017	• Participants sought clarity from primary health unit staff on clinic hours of operation & actions to take if no staff are found at the PHU • Improving data quality & completeness of CHWs’ reporting

*Facility death review*. Case reports compiled following VASA interviews were discussed by a maternal and child mortality review committee in five studies [16, 31, 39, 44, 55]. There was variation in the composition of maternal or child death review committees; some consisted only of health professionals, mostly local doctors, a paediatrician, a gynaecologist, a midwife or nurse, and a pharmacist [31, 32] others included lay health workers or community informants and fieldworkers [31, 39]. After discussing the details of each case, the committee identified avoidable factors, learning needs and proposed actions and resolutions to prevent future deaths. In three studies, the committee reported back to the community its recommendations on measures to prevent future deaths [16, 31, 54].

#### Actionable community responses

Positive outcomes were noted in the five studies that included actionable community responses in Sierra Leone [54], Malawi [31], Mali and Uganda [39], and Bangladesh [16, 55], highlighting dissemination of recommendations to health workers and communities following case review (Table 2).

Involvement of multiple stakeholders including policymakers led to some positive outcomes such as improved availability of data necessary to make programmatic decisions [25, 56], implementation of actionable interventions, enactment of local policies, management and supervision of health facilities and CHW [31, 35, 57, 58]. Participation of local government leaders in social autopsy sessions developed a sense of ownership and commitment to take responsibility for their respective communities [55]. For example, the appointment of district-level MDR leads in Mchinji district, Malawi, improved actionable response of maternal deaths reviews, as 67% of response recommendations were taken up in 2014 compared to 26% in 2013 [58]. One year after the implementation of CDSR in Ethiopia, maternal deaths became the 21^st^ mandatory reportable condition [57].

In Burkina Faso, the Ministry of Health has used data from CDSR to address stockouts of essential commodities for facilities, conducted in-service training for obstetric and newborn care in facilities with high maternal and child mortality rates, and provided surgical equipment to facilities in need [59]. The leadership of Kibaale district in Uganda allocated resources to construct a bridge that helped connect several communities with high mortality rates to the main road and increased access to emergency obstetric care [60]. CDSR in Bangladesh resulted in the implementation of actions following recommendations of the maternal death surveillance and response committee, such as the upgrading of the community clinic to a ten-bed hospital [55].

Death review meetings were shown to be a potentially powerful intervention to enable staff to learn from their shortcomings and to institute significant changes to procedures within their institution [7, 31]. For example, following a review of the death of a woman with eclampsia in Ethiopia, the MDSR committee recommended more rapid consultation with senior staff and purchased a generator and a biochemistry machine. When a similar case presented, all resuscitative measures were conducted with senior attendance by an Emergency Surgical Officer and Gynaecologist [61]. Some of the actions taken following death review meetings to improve staff capacity include implementation of quality improvement efforts, including provision of surgical packs, equipment and drugs; rotation, training and supervision of the surgical team and ensuring availability of transfusion blood from donors for obstetric emergency care [40]. In Uganda, health workers were transferred to under-served areas, and a mobile telephone network was created to fast-track contact with the on-call consultant and paediatrician by nurses and interns [39].

Community members recognised participatory review meetings as a tool to promote preventive messages and a strong basis for collective action to reduce maternal and child mortality, including increased capacity of the community to implement their own action plans and not repeat the same errors that previously resulted in a death [16, 54, 55]. Community participants reported improved trust in the health system in Malawi [31], and the importance of antenatal care (ANC), postnatal care and receiving care at the health facility [53, 55]. In Mali, to avert delays associated with lack of money to access care, some communities established savings groups to provide loans for emergency healthcare, based on community resolutions from participatory death reviews [39].

Pregnant women in Kashipur, Bangladesh, reported that ANC became readily available in the village following the deployment of skilled birth attendants in the community [55]. For example, a 19-year-old woman from Dhormogar village, Bangladesh, committed to delivering her first child by a skilled birth attendant after attending a social autopsy meeting [62]. The CDSR enabled the reduction in annual numbers of maternal deaths from 305 to 163 and neonatal deaths from 3361 to 1670 deaths in 2011 and 2015, respectively, in Bangladesh [63]. In Uganda, after one year of implementation, the Saving Mothers Giving Life initiative, which involved community surveillance and response to maternal deaths, was associated with a 30% reduction in maternal mortality in four districts, from 452 to 316 deaths per 100 000 live births [60].

## Discussion

This scoping review demonstrates that community-based death reporting processes contribute significantly to the identification of maternal and child deaths in settings where CRVS data are not available or incomplete. Our findings show that community-based death reporting processes can be used to supplement maternal and child mortality data in CRVS through integration into the national health management information system, as is already done in India, Pakistan and Bangladesh, contributing to the sustainability and increased capacity of the CRVS systems [7, 44, 56].

Community-based informants (mostly CHWs) were used to identify maternal and child deaths. VA and SA processes were then used to determine the causes and avoidable factors of mortality [31, 50, 56]. Another death identification and reporting method is RAMOS; however, the extent to which this method is practised across LMICs is limited. Utilising CHWs for death identification aligns with the recommended strategy of task shifting in most LMICs.

We found that VA was the most used process for investigating the probable cause of death. The use of VA was previously restricted primarily to research contexts in most African countries, with only a few countries incorporating this method into conventional CRVS systems [43, 50, 64]. Where VA interviews are conducted, they are usually performed by trained health professionals, with only three studies stating the use of CHWs to perform this function. Studies that focused on community death reviews in Malawi, Bangladesh and Uganda trained CHWs to conduct VA interviews and showed that CHWs with at least one year of secondary education can perform VA successfully using simplified questionnaires [31, 50]. Where CHWs did not conduct VA interviews, they reported deaths or validated maternal deaths using RAMOS, following which VA was performed by a trained health professional.

Our findings also showed that VA interviews with the deceased’s next of kin or caregiver can be used to identify the cause of death where no medical cause/s of death would have been assigned. Different tools were used in various settings, but the WHO’s VA tool was most widely used, as it incorporates essential variables and integrates the indicators required for automated diagnostic algorithms [65]. Although such standardisation allows for easy comparison with other standardised methods, (for example, hospital records), having a standardised tool may not capture nuances peculiar to local settings [66]. We suggest that under certain conditions, it may be of value to adapt tools used for VASA to fit the local context.

A common feature in retrospective studies was the long duration of conducting VASA, raising concerns about the accuracy of reports [6, 26, 32]. The WHO recommends that findings from VA methodologies with recalls of more than one year should be interpreted with caution [67, 68]. However, there is no evidence in the current study that studies with a more extended recall period had problems with attributing the cause of death. A study conducted in Bangladesh did not find any evidence that undetermined cause of death was more common for enquiries with a three-year recall period [68]. A study investigating 10882 deaths with complete VAs from the Agincourt Health and Demographic Surveillance System in South Africa showed that a recall period of up to one year did not reduce the reliability of the data or the cause-of-death patterns derived [69]. Some of the strategies recommended for overcoming recall bias include flexibility of interview time and spending enough time with respondents [35, 43, 48].

This review found variation in protocols used to assign causes of maternal and child deaths across studies. Generally, the underlying cause of death was determined by trained physicians with concordance between the reviewers. We also found that community informants were not involved in ascertaining causes of death; a finding supported by Engmann et al. [70] which showed that, despite receiving similar training as physicians, non-physicians were perceived to be unreliable, and thus could not be relied on to determine the underlying cause of perinatal death using VA. Some of our reviewed studies used InterVA, a computer-based algorithm, to establish probable cause of death. InterVA is good at determining disease burdens at a population level when compared to physician review. However, it is much less reliable at determining the specific cause of death of a given individual when compared to physician review [71]. Other computerised algorithms such as Tariff, Simplified Symptom Pattern and Random Forest, were reported to have high accuracy at an individual level compared to physician-certified VA and InterVA in all age groups [49, 71]. However, further research is required to establish the generalisability of Random Forest, Tariff and Simplified Symptom Pattern algorithms in community settings and their compatibility with the WHO VA questionnaire.

Our review shows that CHWs/community informants reported maternal and child deaths to the health facility or district level, and relevant programme staff, on paper, phone call or electronically, depending on the context. Even though necessary quality assurance measures were introduced in CDR processes, there were significant challenges that undermine data quality. This finding compares with the results of a survey in Pakistan which assessed the accuracy of monthly reports submitted by community-based lay health workers and found that only 47.5% of the reports were classified as accurate, while 35% of the reports misreported data and over-reported deaths which did not happen in the catchment area [72]. Earlier studies from Malawi [73] and Ethiopia [74] reported that most of the data collected at the community level and reported to the health facility were incomplete due to management issues, lack of infrastructure, and health policy and politics [75]. The accuracy and completeness of village registers completed by village headmen in Malawi were influenced by insufficient supervision, a high degree of illiteracy of village headmen and non-integration of health facility and village-related data [29]. Use of poor-quality data results in inaccurate information about health systems gaps, which leads to poor management and performance of health programmes [76]. Accurate and complete mortality and health service delivery data reported by CHWs in Mali were attributed to active supervision and incentives (US$30 per month) [36], and by conducting post-interview reflections and debriefings in South Africa [41]. Considering the utility of this data for health planning at district, provincial and national levels, there is a need for adequate supervision, regular auditing and the development of a quality assurance system to improve data quality that will enhance validity and accuracy of community-based maternal and child mortality data. Improving the quality of care of maternal and child health requires the contributions of many actors [31, 54], including the community, civil society organisations, government, and the donor community, with the participation of various stakeholders in community death review meetings [16, 31].

The studies show that CHWs who collected information on deaths played a limited role in discussions on actionable community responses. Community informants ought to be recognised as important collaborators, facilitating the flow of information, as well as agents of engagement between the community, the death review system, health facilities and policymakers. Leadership and commitment from relevant government departments (as shown by the Ethiopian model) formalised and enhanced the collection of reliable and representative cause of death statistics through community-based death review processes [57].

More needs to be done concerning integrating CDSR into national programmes before the full benefit of the community death review process can be obtained in most LMICs. In Sierra Leone, for example, under the government’s National CHW Policy 2016–2020, CHWs have a role in reviewing community health-level data, but mechanisms for this process are yet to be determined [54]. Successful implementation of a CDSR system requires a national-level policy to notify all maternal and child deaths, and a policy to review maternal and child deaths, both in the community and at the facility. There is a need for adequate training and supervision of community informants to enhance the quality of data they report. Also, health workers involved in the reviews should be trained, especially on the action and response cycle, to ensure measures are implemented based on the evidence of the challenges and barriers identified in the CSDR. Further research is required to determine how many deaths need to be investigated and reviewed to maximise impact but minimise the cost of the CSDR system.

## Conclusion

Community-based maternal and child death reporting processes improve the identification of maternal and child deaths in LMICs and subsequent investigation of causes of death and contributing factors. Lay or community health workers are tasked with the identification of deaths, but also conducted VASA in some contexts. With relevant quality assurance measures, it is feasible to fill the gap for accurate statistics on maternal and child mortality and its causes. The measures could include supervision and training of community health workers, use of information technology processes, infrastructure to ensure data accuracy and continuous data, community-based maternal and child death reporting and investigation. Further research is required to develop optimal methods for community death surveillance and review, which maximise impact while minimising cost. Reviewing deaths at community meetings and confidential enquiries can increase understanding of the causes and avoidable factors of maternal and child mortality, and prompt positive action at the local community and health facility to reduce mortality.

## Supporting information

S1 Checklist PRISMA flow diagram of literature search strategy. (DOC)

S1 Table Search strategy. (PDF)

S2 Table Summary of studies included in the review. (PDF)

## Abbreviations

ANC: Antenatal Care
CDSR: Community-Based Death Surveillance and Response
CHWs: Community Health Workers
CRVS: Civil Registration and Vital Statistics
LMICs: Low-and Middle-Income Countries
RAMOS: Reproductive Age Mortality Study
SA: Social Autopsy
VA: Verbal Autopsy
VASA: Verbal Autopsy and Social Autopsy
