# Supplementary material for: Community surveillance and response to maternal and child deaths in low- and middle-income countries: A scoping review
Source: PLoS One. 2021 Mar 16;16(3):e0248143. doi: 10.1371/journal.pone.0248143 (PMC7963102; doi:10.1371/journal.pone.0248143)
Supplement: S1 Table — (PDF) [file pone.0248143.s002.pdf]

S1 Table: Search strategy

Population: Women who died while pregnant or after delivery and children who died under the age of five years

Intervention: Identification and notification of maternal and under-five deaths by a community health worker; and conducting verbal and social autopsy with family members and neighbours in the community to ascertain causes and contributing factors respectively

Comparator: None

Outcome: Maternal and under-deaths identified and investigated

Study design: Any type of study design

Set	Search terms	Hits
1	Verbal autopsy	966
2	Social autopsy	1118
3	Death review	128279
4	Community death review	4123
5	Community-based death surveillance	5152
6	Community notification	39
7	Death audits	44
8	Confidentiality enquiry	426
9	1 OR 2 OR 3 OR 4 OR 5 OR 6 OR 7	134509
10	Maternal deaths	23010
11	Maternal mortality	39368
12	10 OR 11	51075
13	Under-five mortality	1682
14	Under-five deaths	1069
15	Under-5 deaths	1305
16	Infant mortality	115994
17	Infant deaths	79454
18	Child mortality	125412
19	Child death	67626
20	Neonatal deaths	19312
21	13 OR 14 OR 15 OR 16 OR 17 OR 18 OR 19 OR 20	122834
14	12 AND 21	15865
15	9 AND 14	3002
16	Lay Health Workers	278
17	Community Health Workers	23420
18	Lay Interviewers	278
19	16 OR 17 OR 18	25360
20	15 AND 19	72
