# Supplementary material for: Community surveillance and response to maternal and child deaths in low- and middle-income countries: A scoping review
Source: PLoS One. 2021 Mar 16;16(3):e0248143. doi: 10.1371/journal.pone.0248143 (PMC7963102; doi:10.1371/journal.pone.0248143)
Supplement: S2 Table — (PDF) [file pone.0248143.s003.pdf]

S2 Table: Descriptive information for each study included in the scoping review

Author (Year); Country	Definition of mortality measures	Direction of enquiry	Recall period	Data collection tool	Framework for classification of avoidable factors of death	Quality assurance method	Effects	Causes of death and avoidable factors	Limitations and challenges
Negandhi et al., [56]<br><br>India	All women who die within nine months of registering for antenatal care or within 2 months of registering for postnatal care.<br><br>The standard case definition of the WHO used to categorise perinatal deaths	Prospective	Not specified	Not specified	Not specified	Reporting from multiple sources reduced the likelihood of missed deaths	Review system facilitated supportive supervision<br><br>Provided village level data needed to make programmatic decisions	-	Review system facilitated supportive supervision<br><br>Provided village level data needed to make programmatic decisions
Dikid et al., [25]<br><br>India	Not reported	Prospective	Not specified	Not specified	Pathway analysis; Three delays model	-	Inclusion of multiple stakeholders including policy makers has resulted in greater attention to issues that were not solely in	Haemorrhage was the most common cause of death<br><br>Eclampsia was the second most common cause of death followed by sepsis	MAPEDIR model in its present form was found not to have clearly defined channels for disseminating information to various stakeholders

							the domain of the health sector	54% of the delays could be attributed to delay in deciding to seek care for an obstetric complication<br><br>30% delay in coordinating transport<br><br>16% delay in obtaining care	
Rai et al., [42]<br><br>India	Not reported	Prospective	2-6 weeks	Ballabgarh VA tool; INDEPTH-WHO Social autopsy too	Three delays model; Pathway analysis	Randomly selected households were revisited by health supervisors and medical doctors	-	Most common cause of neonatal deaths was birth asphyxia (31.5%), low birth weight (26.5%) sepsis or pneumonia (16.9%), congenital anomaly (9.3%)<br><br>Among post-neonatal deaths pneumonia (28.9%), other	-

								infection and sepsis (13.6%); diarrhoea (15.3%)<br><br>Delay at any level was observed in 50% of neonatal deaths and 41% of post-neonatal deaths	
Singh et al., [44]<br><br>India	Not reported	Prospective	3 weeks	The government of India standardised verbal autopsy tool	Three delays model	Senior public health officer regularly sensitized staff at peripheral centres regarding importance of listing every female death and suspected maternal deaths	-	-	Underreporting of abortion-related maternal deaths especially illegal or in early antenatal period could be missed<br><br>No reporting from primary health centres was observed which staff attributed to fear of punitive action
Biswas et al., [7]<br><br>Bangladesh	Not reported	Prospective	Not specified	Verbal autopsy questionnair e	Not specified			Family had delayed in decision making to seek treatment before death<br><br>There was delay in decision	Presence of community leaders biased discussions

								making to transfer the mother immediately to referral facility<br>Majority of the mothers were found with high blood pressure, blurring vision, swelling of the face and leg<br><br>Majority of mothers had bleeding	
Biswas et al., [16]<br><br>Bangladesh	Not reported	Prospective	15-30 days	Social autopsy tool	Not specified				
Biswas et al., [55]<br><br>Bangladesh	Not reported	Prospective	Not specified	A structured death notification form	Not specified	Number of deaths, especially maternal deaths fell in Kashipur<br><br>Upgrading of the community clinic to a ten-bed hospital - based on MDSR committee recommendations<br><br>ANC is now easily available in the village - a trained community skilled birth attendant was deployed to perform			No comparison was made with a geographic area where there is no MNDR

						antenatal care, normal delivery, and postnatal care at the facility			
Halim et al., [37] Bangladesh	The standard case definition of the WHO	Prospective	15-60 days	WHO-based verbal autopsy tool	Three delays model	Supervisor accompanied health workers conducting VA and provided instant feedback<br><br>VA forms were reviewed monthly by supervisors and central investigators<br><br>Family was revisited in case of any inconsistences to clarify any identified issues			
Soofi et al., [9]<br><br>Pakistan	The standard case definition of neonate's deaths	Prospective	2-6 weeks	WHO-neonatal verbal autopsy tool;<br>Clinical case sheets	Not specified	A 2-day refresher training for CHWs every 6 months<br>Review meetings<br>Supervisory field visits to ensure adequacy of VA procedures<br>Random 5% of verbal autopsy interviews were attended by the study supervisor to ensure interview procedure and probing			

						techniques were being applied<br>2% work of each CHW was verified by a blinded social scientist to ensure that data collected by the CHW is correct			
Mir et al., [26]<br><br>Pakistan	WHO standard case definition	Retrospective	2 years	WHO VA questionnaire	Not specified	-	-	Obstetric haemorrhage was the leading cause of death<br>40% of deaths due to pregnancy induced hypertension<br>5% of deaths were due to abortion related complications	Misclassification; village-based informants had incorrectly classified 3 deaths out of 168 pregnancy-related deaths<br><br>18 of the 169 deaths were identified as eligible pregnancy related deaths when they were false matches
Bogale et al., [6]<br><br>Ethiopia	Not reported	Retrospective	18 months	INDEPTH Network Social autopsy tool	Three delays model	-	-	Birth asphyxia and bacterial sepsis were the leading causes of death contributing 32.5% of	Affected by misclassification bias that might be introduced when trying to differentiate neonatal deaths

								deaths, followed by prematurity which contributed 14%<br><br>For neonates between 1 and 6 and 7-28 days, the major cause of death was bacterial sepsis accounting for 44.4% and 66.7% respectively	from stillbirths during the verbal autopsy A long recall period of 18 months introduces recall bias
Willcox et al., [39]<br><br>Mali & Uganda	Not reported	Prospective	Within 4 weeks	QUARITE trial questionnair e	Not specified	Re-investigated a random sample, cross checking with other sources of information where possible	-	Malnutrition in Mali<br><br>Child neglect in Uganda	Difficult to follow up implementation of recommendatio ns made to address social factors of child death discussed in village meetings
Moshabela et al., [40]<br><br>Senegal	WHO standard case definition	Prospective	1-2 weeks	MVP standardised VASA tool based on	Pathway analysis	Childcount+ platform with built in reminders was used for data collection, and		Four of the deaths occurred as a result of haemorrhage	Small number of deaths identified pose a challenge in

				WHO questionnaire		monitoring of CHW workload and performance<br><br>Periodic retraining of CHWs was conducted to optimize accuracy of data collection<br><br>Clarification was sought from CHWs, clinic staff or household members if required	-		calculating Maternal Mortality Ratio
Bayley et al., [31]<br><br>Malawi	WHO standard case definition	Prospective	Not specified	Verbal autopsy form	Three delays model	-	CLMDR process doubled the number of maternal deaths being reviewed with 86% identified maternal deaths being reviewed<br><br>The process resulted in high rates of completion of community-planned actions (82%), district hospital (67%)	-	System struggled to identify or follow-up maternal deaths of transient workers due to reduced coverage<br><br>Verbal and social autopsy was not always able to facilitate discussion of sensitive topics including

							and health centre (65%) actions to prevent maternal deaths<br><br>Community participants reported improved trust in the health system, with potential benefits for uptake of available healthcare		abortion or HIV related deaths<br><br>It requires increased staff attendance at health facility CLMNDR meetings<br><br>System relies on CHWs, who have a lot of other responsibilities, to link health services and the community<br>Were HSAs failed to identify families or organise meetings, the process failed
Adomako et al., [32]<br><br>Ghana	WHO standard case definition for pregnancy-related deaths	Retrospective	5 years	RAMOS interview record sheets<br>VA form - Ghana Health Service &	Not specified				Family members only identified 48% of maternal deaths correctly

				WHO recommendations					
Mgawadere et al., [27]<br><br>Malawi	WHO standard case definition	Prospective	30 days	WHO Verbal autopsy tool	Not specified	Research staff visited all 46 participating healthcare facilities once a month, cross checked all registers and checked findings with the respective healthcare providers<br><br>Quarterly review meetings were held with health surveillance assistants to identify any death not reported	The study identified an additional 8 maternal deaths which had occurred at facility level which had not previously been reported and which had occurred in wards other than the maternity ward	Obstetric haemorrhage was the leading cause of death accounting for 47.8%, followed by pregnancy related infections 19.4%, hypertensive disorders in pregnancy, childbirth and the puerperium 16.8% and pregnancy with abortive outcome 13.2%	It was not possible to conduct a verbal autopsy for all deaths of women of reproductive age<br><br>Reliance on reported symptoms of pregnancy in case-notes, registers and via relatives of the deceased, means that some women with undisclosed or undiagnosed pregnancy as well as those were signs and symptoms were simply not reported/or documented

									could have been missed
Mgawadere et al., [28]<br><br>Malawi	WHO standard case definition	Prospective	Not specified	WHO Verbal autopsy tool	Three delays model	Research staff visited all 46 participating healthcare facilities once a month, cross checked all registers and checked findings with the respective healthcare providers<br><br>Quarterly review meetings were held with health surveillance assistants to identify any death not reported	-	94.7% of women had had delayed treatment on admission Shortage of equipment, drugs and supplies was the second most frequent cause of type 3 delays (63.1%)<br><br>20 out of 28 maternal deaths were associated with healthcare provider factors which were avoidable and administrative failure	-

Zaba et al., [52] Multi-country study	All deaths in pregnant or postpartum women (up to 42 days postpartum) – did not exclude cases that are incidental to pregnancy)	Prospective	Not specified	WHO Verbal autopsy tool	Not specified	-	Of the 235 pregnancy related deaths, 40(17%) were identified as pregnancy related by both VA data and demographic surveillance data<br><br>144 (61.3%) were identified as pregnancy-related based on VA reports alone, and the remaining 51 (21.7%) were identified through demographic surveillance only)	-	27.6% of the data in the dataset did not have an associated VA, might have missed some pregnancy related deaths<br><br>Studies that undertake demographic surveillance with intervals longer than 6 months would not intersect with all times during which women would recognise that they are pregnant

Kakoty et al., [46]<br><br>India	Definition of neonatal mortality not specified	Prospective	VA conducted within a month of reported death	Predesigned pretested VA questionnair e	Not specified	Not specified	Not specified	Pneumonia and septicaemia accounted for 47.7% of death followed by asphyxia and respiratory distress syndrome. 25% of newborn babies had LBW or prematurity. Complicated or prolonged labour was reported in about 46 % deliveries. Traditional beliefs influence health seeking behaviour	Not specified
Gupta et al., [47]<br><br>Rwanda	Not specified	Retrospectiv e	Three weeks to one year following the child's death	2012 WHO VA tool	Not specified	Data was collected from multiple sources	Not specified	About 33% of the neonates did not visit a hospital during their illness and 39.9% did not receive treatment for the illness that	Accuracy of InterVA4 algorithm in identifying cause of death especially for neonates

								preceded their death<br><br>Major causes of death were pneumonia and birth asphyxia among neonates, and among non-neonates - malaria, acute respiratory infections and HIV/AIDS	
Roder-DeWan et al., [45]<br><br>Rwanda	Child mortality: Deaths of children aged 1-5 years	Prospective	3 weeks-1 year after the child's death	WHO 2012 VA tool	Three delays model<br><br>Lancet Global Health Commission on<br>High Quality Health Systems framework	Used a community-based triangulation method to capture the most marginalised families and to identify those that have left the catchment area after a child's death<br><br>Regular debriefings during data collection and processing	Not specified for CHWs	Leading cause o death was malaria (39%), respiratory illness (14%) and acute abdomen (14%)<br><br>Caregivers did not adhere to treatment plans	Not specified for CHWs

Kallander et al., [51]<br><br>Mozambique	Deaths in children 2-59 months	Prospective	Four months	WHO VA tool<br><br>INDEPTH Network SA tool	Pathway to survival	Structured regular supervision in the field throughout the data collection period, and all questionnaires were reviewed by the interviewers and their supervisors separately on a daily basis	Not specific to CHW notification	80% of deaths occurred at home; lack of caregiver awareness and recognition of illness symptoms<br><br>Major causes of death are malaria (50.6%); HIV/AIDS related deaths (11.8%); diarrhoeal diseases and acute respiratory infections	Not specific to CHW notification
Nabukalu et al., [50]<br><br>Uganda	Not specified	Prospective	Not specified	WHO 2014 VA questionnair e	Not specified	20% of the VA interviews performed by CHWs were directly observed by the trainers<br><br>Inspection of completed questionnaires	Not specified	Leading causes of death wee malaria (19.5%), prematurity (19.5%), neonatal pneumonia (15.6%),	Not specified

Hutain et al., [35]<br><br>Sierra Leone	Neonatal deaths: deaths during 28 days of life	Prospective	6 months	WHO 2007 VA Questionnaire; Population Health Metrics Research Consortium Shortened Questionnaire; WHO 2014 VA Questionnaire	Not specified	Not specified	Sharing verbal autopsy findings increased care-seeking behaviour	Acute respiratory infections, malaria and neonatal conditions	Delays in data collection and capturing of CHW reports into the electronic vital registration systems<br><br>Incomplete data submitted by CHWs
Singogo et al., [29]<br><br>Malawi	Not specified	Retrospective	Not specified	Structured proforma	Not specified	Village headmen birth registers were verified with facility records of births	Not specified	Not specified	Incompleteness of village health registers (66% were nearly 95% of data entered<br><br>Insufficient supervision to ensure proper and complete documentation of vital events; no system in place to reconcile health facility and

									village-related data<br><br>High degree of illiteracy in village headmen
Joos et al., [33]<br><br>Malawi	Not specified	Prospective	Not specified	Village Health Register	Not specified	Supervisors visited HSAs in their catchment area each month to check their extraction forms along with the registers<br><br>Data quality guidelines reinforced during biannual data review meetings<br><br>Household surveys conducted to assess the validity of the events reported by HSAs	Not specified	Not specified	Community based real time monitoring system did not accurately capture either the levels of, or trends in, childhood mortality. Health surveillance assistants underestimated under-five deaths by 49%

Munos et al., [36]<br><br>Mali	Not specified	Prospective	Not specified	Pregnancy, birth and death register	Not specified	Monthly field visits to supervise the lay health volunteers<br><br>Verification of a sample of 10% sample of reported births and deaths in randomly selected village health registers	The community-based model in Mali produced estimates of the under-five mortality rate that were equivalent to those produced by the full pregnancy history	Not specified	Highly nomadic population<br><br>Follow-up on pregnancy outcomes was not done in a systematic manner
O'Connor et al., [54]<br><br>Sierra Leone	Children who die before reaching the age of five years	Prospective	Not specified	Standardised Ministry of Health materials	Not specified	Community discussions to improve CHW functionality	Communities meetings formulated strategies to enhance CHW motivation as well as quality and completeness of CHW monthly reports	Not specified	Underreporting of morbidity data and vital events by CHWs<br><br>Low rate of CHW report submission
Amouzou et al.,[34]<br><br>Malawi	Not specified	Prospective	Not specified	Village Health Register	Not specified	Supervisor checked the HSA's performance in completing the extraction forms and recording the births and deaths in the village health register Supervisors provided on-the spot retraining and feedback	Not specified	Not specified	Under-estimation of under-five mortality<br><br>Ensuring regular supervision of HSAs

Study	Perceptions and acceptance	Challenges
Biswas et al., [62]	Social autopsy added a new dimension to the work of health workers<br><br>SA allowed the community to explore their own barriers and develop solutions<br><br>SA was highly accepted at the community level since the community recognised SA as a powerful tool to promote preventative messages at the optimal time<br><br>Local government leaders participating in SA sessions developed a sense of ownership and commitment to take responsibility for their respective community	Challenges in involving more male participants, since in most cases they are the decision makers
Abebe et al., [57]	Clear messages from the Ministry imbued MDSR with a sense of prioritisation and urgency and thus respondents felt obliged to deliver MDSR as part of national plans<br><br>Leadership at lower levels increased pressure for results<br><br>Integration of MDSR inti PHEM was seen to pool strengths from both teams and increase the likelihood of follow-up action<br><br>At aggregate level, district, zonal or regional committees were seen to have increased their capacity to assess local patterns in maternal mortality<br><br>Having reliable data on which to act, has led to improvement in documenting and managing case notes<br><br>Availability of more detailed information strengthened communication across the health	Smooth introduction of MDSR was impeded by widespread fear that an increase in reported maternal deaths could lead to legal or disciplinary action<br><br>There was still lack of clarity around how the two departments should work together

	system, as well as between individual health providers, and between health authorities and communities	
